# Transcription Profiling Reveals Potential Mechanisms of Dysbiosis in the Oral Microbiome of Rhesus Macaques with Chronic Untreated SIV Infection

**DOI:** 10.1371/journal.pone.0080863

**Published:** 2013-11-29

**Authors:** Susan Ocon, Christina Murphy, Angeline T. Dang, Sumathi Sankaran-Walters, Chin-Shang Li, Ross Tarara, Niku Borujerdpur, Satya Dandekar, Bruce J. Paster, Michael D. George

**Affiliations:** 1 Department of Medical Microbiology and Immunology, University of California Davis, Davis, California, United States of America; 2 Department of Microbiology, Forsyth Institute, Cambridge, Massachusetts, United States of America; 3 Department of Microbiology, Immunology, and Molecular Genetics, University of California Los Angeles, Los Angeles, California, United States of America; 4 Department of Public Health Sciences, University of California Davis, Davis, California, United States of America; 5 California National Primate Research Center, University of California Davis, Davis, California, United States of America; 6 Department of Oral Medicine, Infection and Immunity, Harvard School of Dental Medicine, Boston, Massachusetts, United States of America; Emory University School of Medicine, United States of America

## Abstract

A majority of individuals infected with human immunodeficiency virus (HIV) have inadequate access to antiretroviral therapy and ultimately develop debilitating oral infections that often correlate with disease progression. Due to the impracticalities of conducting host-microbe systems-based studies in HIV infected patients, we have evaluated the potential of simian immunodeficiency virus (SIV) infected rhesus macaques to serve as a non-human primate model for oral manifestations of HIV disease. We present the first description of the rhesus macaque oral microbiota and show that a mixture of human commensal bacteria and “macaque versions” of human commensals colonize the tongue dorsum and dental plaque. Our findings indicate that SIV infection results in chronic activation of antiviral and inflammatory responses in the tongue mucosa that may collectively lead to repression of epithelial development and impact the microbiome. In addition, we show that dysbiosis of the lingual microbiome in SIV infection is characterized by outgrowth of *Gemella morbillorum* that may result from impaired macrophage function. Finally, we provide evidence that the increased capacity of opportunistic pathogens (e.g. *E. coli*) to colonize the microbiome is associated with reduced production of antimicrobial peptides.

## Introduction

A majority of HIV-infected individuals world-wide do not have adequate access to antiretroviral therapy and ultimately experience opportunistic oral infections [1]–[4] that are linked to deterioration of immune function [5], [6] and severely impact nutritional health [7], [8]. Despite the global impact, surprisingly little is known about the mechanisms that drive HIV-associated pathogenesis of the oral mucosa. In healthy subjects, oral infections are prevented through physical barriers supplied by the epithelium and its commensal microbiota [9]–[12] and by rapid and robust mobilization of innate immune responses [13]–[15]. Commensal bacteria that colonize each of the numerous unique microenvironments within the oral cavity have co-evolved as homeostatic communities that facilitate regulatory, healthy immune interactions [13], [16], [17]. Thus, the various types of oral manifestation of HIV disease are likely to be connected to profound yet potentially unrelated perturbations (dysbiosis) of distinct, extremely complex and delicately balanced host-microbe ecosystems.

Although studies of oral microbial dynamics can be readily accomplished in HIV patients through non-invasive sampling [18], [19], investigations that utilize a systems biology approach to explore mechanisms of dysbiosis are impractical to implement in humans due to constraints in patient numbers and sample collection. Rhesus macaques infected with simian immunodeficiency virus (SIV) have the potential to circumvent such restrictions, and are already well-established as an animal model for the immunopathologies of HIV disease; displaying CD4+ T cell depletion kinetics [20], [21] and mucosal pathologies [22], [23] strikingly similar to humans. Although the oral microbiota of rhesus macaques has not been characterized to date, the anatomical and the physiological analogies between macaques and humans imply that their microbiomes perform the same relative functions. Therefore, SIV infected macaques are likely to provide a valuable, yet untapped, resource for investigating mechanisms of oral mucosal immunopathology, host-microbe dysbiosis, and manifestation of secondary infections.

In the current study, we seek to initiate development of the rhesus macaque model of oral pathogenesis in SIV infection. We provide the first characterization of the rhesus macaque oral microbiome, and utilize high throughput gene expression profiling to evaluate mechanistic relationships between SIV-induced oral pathogenesis, host immune status, and modulation of the lingual (tongue) microbiota. Our findings indicate that untreated SIV infection results in constitutive induction of antiviral and inflammatory responses in the lingual mucosa, the latter of which is characterized by dysregulated cytokine production that may exclusively promote interferon gamma (IFNγ) driven responses. Comparisons of *in vivo* gene expression in the tongue epithelium of SIV infected animals with *in vitro* expression profiles of oral epithelial cells treated with IFNγ provide evidence that chronically activated IFNγ signaling may contribute to dysbiosis by impairing antimicrobial peptide production and compromising barrier development. In addition, we report a potential link between down-modulation of genes that mediate macrophage functions and a dramatic outgrowth of *Gemella. morbillorum* on the tongue dorsum during SIV infection.

## Materials and Methods

### Ethics Statement

The study was carried out in strict accordance with the recommendations in the Guide for the Care and Use of Laboratory Animals of the National Institutes of Health. The protocol was approved by the Institutional Animal Care and Use Committee (IACUC) of the University California, Davis (Protocol #16648). All efforts were made to minimize suffering.

### Animal groups and welfare, SIV infection, and sample collection

A total of 16 male adult rhesus macaques from the California National Primate Research Center (CNPRC) were utilized for the study. Animals were housed in accordance with American Association for Accreditation of Laboratory Animal Care standards, and experiments were designed to adhere to the “Guide for the Care and Use of Laboratory Animals” of the Institute of Laboratory Resources, National Resource Council. Animals used in SIV experiments were housed indoors, carefully paired to facilitate daily social contact, and provided multiple environmental enrichments to promote psychological well-being. Indoor-living animals were fed a commercial monkey chow (Purina, Inc.) supplemented with seasonal fruits and vegetables. Animals used for tongue swab samples and dental plaque scrapings were from naturally formed family groups living outdoors in an enclosed free-range setting with natural vegetation. Outdoor-living animals were fed commercial monkey chow supplemented with fruits and vegetables, occasional peanuts and sunflower seeds, and grass clippings, small branches, insects, and soil that are consumed during their normal daily activities.

SIV infected animals were observed on a daily basis by veterinary technicians, and CNPRC veterinarians using established protocols performed all procedures. All possible efforts were made to minimize animal pain and discomfort. Ketamine was given intramuscularly (10 mg/kg) when it was necessary for animals to be immobilized. Consistent with the recommendations of the Panel on Euthanasia of the American Veterinary Medical Association, animals in SIV studies were euthanized by overdose of sodium pentobarbital (60 mg/Kg I.V.).

16S phylogenetic analyses were performed on lingual epithelial brushings (n = 3) and dental plaque scrapings (n = 5) collected from free-range living animals, and archived lingual tissues collected during previous SIV studies (3 uninfected controls; 5 SIV infected). Archived lingual tissues were also utilized for gene expression analyses and immunohistochemistry. SIV infected animals were inoculated intravenously with 100_TCID50_ of SIVmac251 as previously described [24]. Longitudinal blood samples were collected prior to SIV infection and at 2 and 10 weeks post-infection (p.i.) from macaques 34967, 34995, 35470, and 35804, and at 2, 10, 12, 14, 16, 18, 20, and 22 weeks p.i. from macaque 34030 (necropsied at 23 weeks p.i.), and utilized for analyses of SIV burden and T cell subset profiles.

### DNA extraction, 16S amplification, cloning and sequencing

DNA was extracted from dorsal tongue epithelial brushings, plaque scrapings, and necropsied tongue tissue samples with the Epicentre® MasterPure Complete DNA and RNA Purification Kit (Epicentre) according to previously published protocols [25]. Genes encoding 16S rRNA (rDNAs) were amplified with a commercially synthesized (Operon Technologies) universal primer set (forward primer, 5′-GAG AGT TTG ATY MTG GCT CAG; reverse primer, 5′- GAA GGA GGT GWT CCA RCC GCA). The resulting PCR product (approximately 1500 base pairs) was identified by electrophoresis and purified from a 1% agarose gel. Cloning of PCR amplified DNA was performed using a TOPO TA Cloning Kit (Invitrogen) following the manufacturers' instructions. Purified PCR amplified DNA was then sequenced using an ABI prism cycle-sequencing kit and an ABI 9700 system (Applied Biosystems, Inc.).

### Phylogenetic analysis of 16S rRNA

Analysis of 16S rRNA encoding sequences was performed as previously described [26]. Briefly, approximately 500 base pair sequences were first utilized to determine identity or approximate phylogenetic position. Full sequences (about 1,500 bases) were then analyzed for each potentially novel species. To identify closest relatives, sequences of the potentially novel organisms were compared to the 16S rRNA gene sequences of over 30,000 microorganisms in the Human Oral Microbiome Database [27] as well as sequences in GenBank and the Ribosomal Database Project. To be included, the taxon must have been detected more than once and have a near full-length (>1450-base) 16S rRNA gene sequence less than 98.5% similar to previously defined taxa. Sequences named for a species were greater than 98.5% similar to that of the type strain of the species. Whenever multiple sequences were available in GenBank for a species, the longest and most accurate was chosen to represent the species.

### TR146 oral epithelial cell cultures

TR146 oral epithelial cells were a generous gift from Cancer Research UK [28]. Cells were grown in two 6-well culture dishes (NuncBrand) in DMEM/F12 media (Invitrogen) supplemented 10% FBS (Gemini), and 1% antibiotic/antimycotic (Gibco). To determine the effects of IFN gamma on epithelial development, cells were grown to ∼80% confluence before replacing the media with media supplemented with 20 mM IFN gamma (GenScript) or, in mock-treated cultures (buffer only). Cells were then incubated at 37°C for 24 hr. before harvesting by centrifugation for downstream gene expression analysis. Six independent experiments utilizing IFN gamma-treated and mock-treated control cultures were performed and the RNA extracts from each were pooled prior to microarray processing.

### RNA extraction and transcriptional profiling by microarray

Total RNA was extracted from lingual whole tissue samples and TR146 cells utilizing protocols and reagents in the Qiagen RNeasy© RNA isolation kit. Messenger RNA amplification, labeling, hybridization to Rhesus macaque-specific GeneChips© (Affymetrix) staining and scanning were performed as previously described [29] utilizing reagents and protocols described in the Affymetrix Gene Expression Analysis Technical Manual. RMA-based, (GeneSpring©) algorithms were utilized to identify differentially expressed genes (DEG) between uninfected controls and SIV infected animals. A minimum fold-difference of +/−1.5 (*p*-value ≤0.05) was used as cut off criteria for generating DEG lists for whole tissue analysis and +/− 1.2 for analysis of epithelial cells isolated by laser capture microdissection. DEG lists were hierarchically clustered and sub-clusters were subjected to pathway analysis using Ingenuity Pathway Analysis (IPA©) and DAVID (http://apps1.niaid.nih.gov/david) web-interfaced software. Microarray data files are deposited at the Gene Expression Omnibus (http://www.ncbi.nlm.nih.gov/geo) under the following accession number: (GSE51445).

### Cell isolations by laser capture microdissection

Oral epithelial cells were isolated from tongue tissues of SIV infected and uninfected control animals by laser capture microdissection (LCM). Prior to LCM, fresh frozen tissues embedded in OCT were cut into 5 µm serial sections and briefly stained with hematoxylin and eosin. Approximately 5×10^3^ oral epithelial cells from each section were microdissected using an Arcturus XT© LCM system (Arcturus) and total RNA was immediately extracted using a PicoPure© RNA extraction kit (Arcturus) and utilized for transcriptional profiling by microarray.

### Quantitative RT- PCR

Real-time-PCR (RT-PCR) analyses were used to validate microarray findings by comparing mRNA levels of tetherin (BST2), myxovirus protein-1 (MX1), signal transducer and activator of transcription-1 (STAT1), interferon gamma (IFNγ), interferon alpha (IFNα), tumor necrosis factor alpha (TNFα), and interleukin-1 beta (IL1β). RT-PCR was carried out on a Viia 7 Real-Time PCR system (Applied Biosystems, Inc.) and data was analyzed with the Viia 7 software package. Internal normalization of C_T_ values was performed based on expression of GAPDH and differential gene expression was determined using previously published protocols [30] by comparison of mean C_T_ values from SIV infected groups and healthy uninfected controls. Statistical analyses (Student's t-test) were used to determine if differences in the mean values for uninfected and infected groups were significant (p-value<0.05).

### Immunohistochemistry

Monoclonal antibodies to human psoriasin (Santa Cruz Biotechnology) were utilized to identify and localize macaque psoriasin by fluorescent immunohistochemistry in 5 µm lingual tissue sections from SIV infected macaques and uninfected controls utilizing previously published protocols [31]. Briefly, psoriasin was visualized following incubation with Cy3-conjugated anti-mouse secondary antibodies. Tissue sections were incubated with DAPI (Invitrogen) to visualize the nucleus and images were captured by confocal laser microscopy using an LSM 5 system and PASCAL software (Zeiss).

### Viral load measurements

SIV RNA copies were detected by quantitative RT-PCR in the blood of infected macaques utilizing previously published protocols and target probes designed to hybridize with the gag region of SIVmac251 [29]. Briefly, a standard curve for SIV RNA copies was first generated with serial dilutions of SIV-infected tissue culture supernatants containing cell-free SIV. RNA isolated from plasma was then amplified by RT-PCR and corresponding C_T_ values were extrapolated against the standard curve to quantitate viral RNA copies/ml of plasma.

### Analysis of T cell subsets in blood

CD4+ and CD8+ T cell percentages in the peripheral blood were determined by flow cytometry at the indicated time points. Briefly, Ficol-gradient isolated PBMC were stained with fluorochrome labeled monoclonal antibodies to CD3, CD4, and CD8 (Santa Cruz Biotechnology). Negative controls were stained with isotype control monoclonal antibodies. Fluorescence was measured on a LSRII flow cytometer (Becton Dickinson) and FlowJo (Treestar) was utilized to analyze the data. Student's t-tests were utilized to determine if differences in the mean percentage of T cells in each subset were significant (p-value<0.05) between SIV infected animals and controls.

## Results

### Characterization of the rhesus macaque oral microbiota

**Figure 1:**
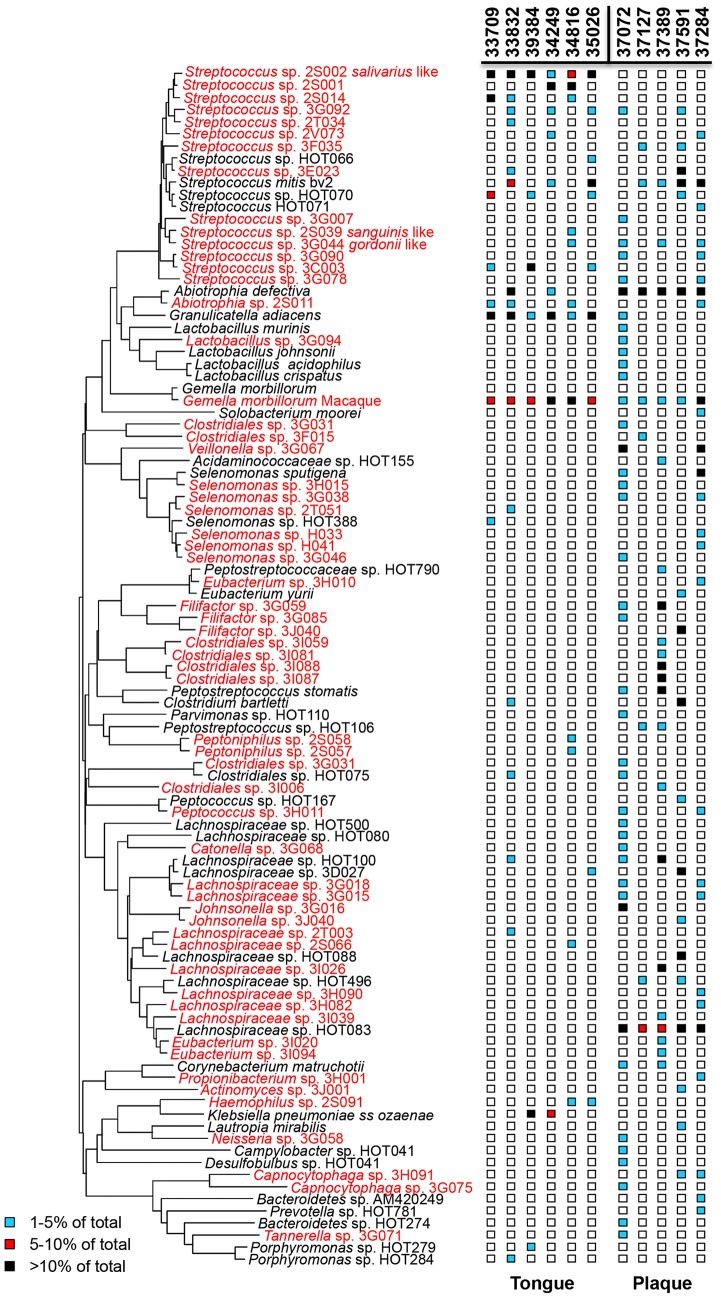
Neighbor-joining tree for rhesus macaque tongue and dental plaque taxa. The oral taxon number follows the name of each taxon. Bacterial species common to the human oral microbiome are shown in black text and species unique to the macaque in red.

In implementing a systems biology approach to investigate the relationship between SIV associated oral pathogenesis and host-microbe dysbiosis, we first sought to characterize the types of bacterial species that colonize the rhesus macaque oral microbiome. Lingual mucosal (nylon brush swabs or homogenized tissue) samples and dental plaque scrapings were collected from 11 healthy adult male macaques housed at the California National Primate Research Center (CNPRC). Clonal analysis of 16S rRNA genes revealed that the rhesus macaque oral microbiome harbors many of the same bacterial species that colonize the human oral cavity as well as numerous novel species that appear to be “simian counterparts” of human oral bacteria (Figure 1). Consistent with reports of the human oral microbiome [26], the bacterial community profiles of the macaque lingual and dental microenvironments were clearly distinct, and substantial variability was detected in species diversity (from 4 to 41 species) among individuals. Overall, 128 bacterial species from 32 genera were identified on the tongue and/or in dental plaque, of which 82 (64%) appear to be novel, and the remainder (36%) corresponding to human oral bacteria. Notably, although the bacterial species that were identified as “macaque-specific” are presumably unique, were not substantially divergent from human commensal species.

**Figure 2:**
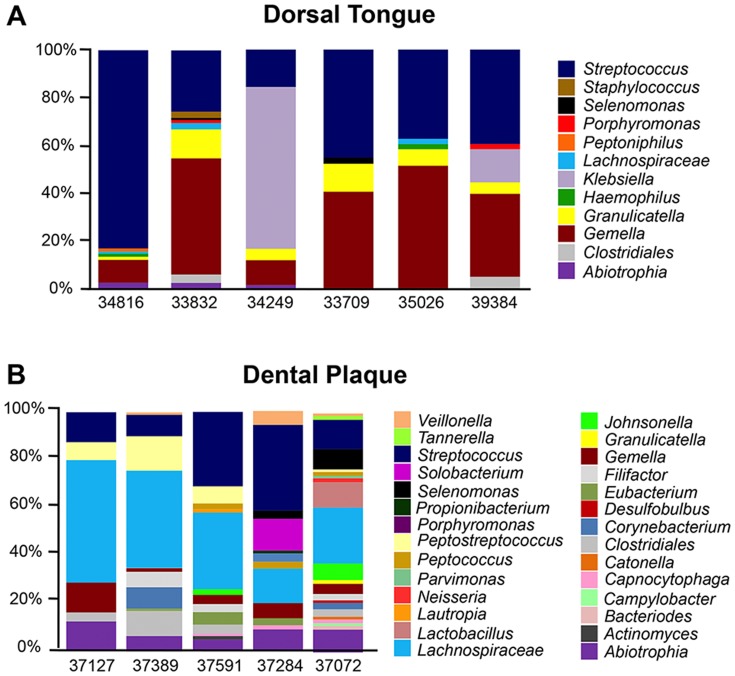
Phylogenetic distribution of bacteria on the tongue and in dental plaque of healthy macaques. Abundance distribution of 32 different bacterial genera identified A) on the tongue dorsum, and B) in the dental plaque of healthy uninfected rhesus macaques.

As reported in humans [32], *Streptococcus* was one of the predominant commensal genera colonizing the lingual epithelium of healthy uninfected macaques, comprising from 15 to 83% of the total community in the animals that were tested (Figure 2A). Human-colonizing *S. mitis*, *S. australis*, and *S. oralis* and *S.salivarius* species were also identified as well as macaque-specific *S. oralis*-like, *S. salivarius*-like, *S. peroris*-like, *S. sanguinis*-like, *S. gordonii*-like, and *S. parasanguinis*-like species. *Gemella* (both human-colonizing and macaque-specific) was either the most or the second most abundant genera detected in all but one animal, comprising from 8% to 40% of the total bacterial population. All of the healthy macaques tested also harbored human-colonizing *Granulicatella adiacens* as 1 to 10% of the total lingual bacterial community. In addition to three core genera, *Streptococcus, Gemella*, and *Granulicatella*, 50% of healthy macaques (3 of 6) showed colonization of *Lachnospiraceae*. *Abiotrophia, Haemophilus*, and *Klebsiella* were also detected in 33% (2 of 6) of the samples. Notably, each of the 6 genera identified on the tongue dorsum of at least 2 animals are also found as commensals on the human tongue.

In contrast to the tongue dorsum, the core bacterial taxa identified in macaque dental plaque included *Streptococcus* and *Lachnospiraceae*, with substantial but comparatively less proportions of *Gemella* and *Abiotrophia* (Figure 2B). While the similarity in the proportion of these genera between macaques and humans requires further study, each has been identified in multiple studies as a commensal component of human dental plaque [26], [33]. Thus, taken together, the parallels in bacterial genera inhabiting the dental plaque and tongue dorsum of macaques to that of humans confirm that their microbiomes are likely to perform equivalent functions, and reveal a new potential role for macaques as a highly relevant non-human primate model for a variety of human oral diseases.

### Modulation of the lingual microbiome in untreated chronic SIV infection

**Figure 3:**
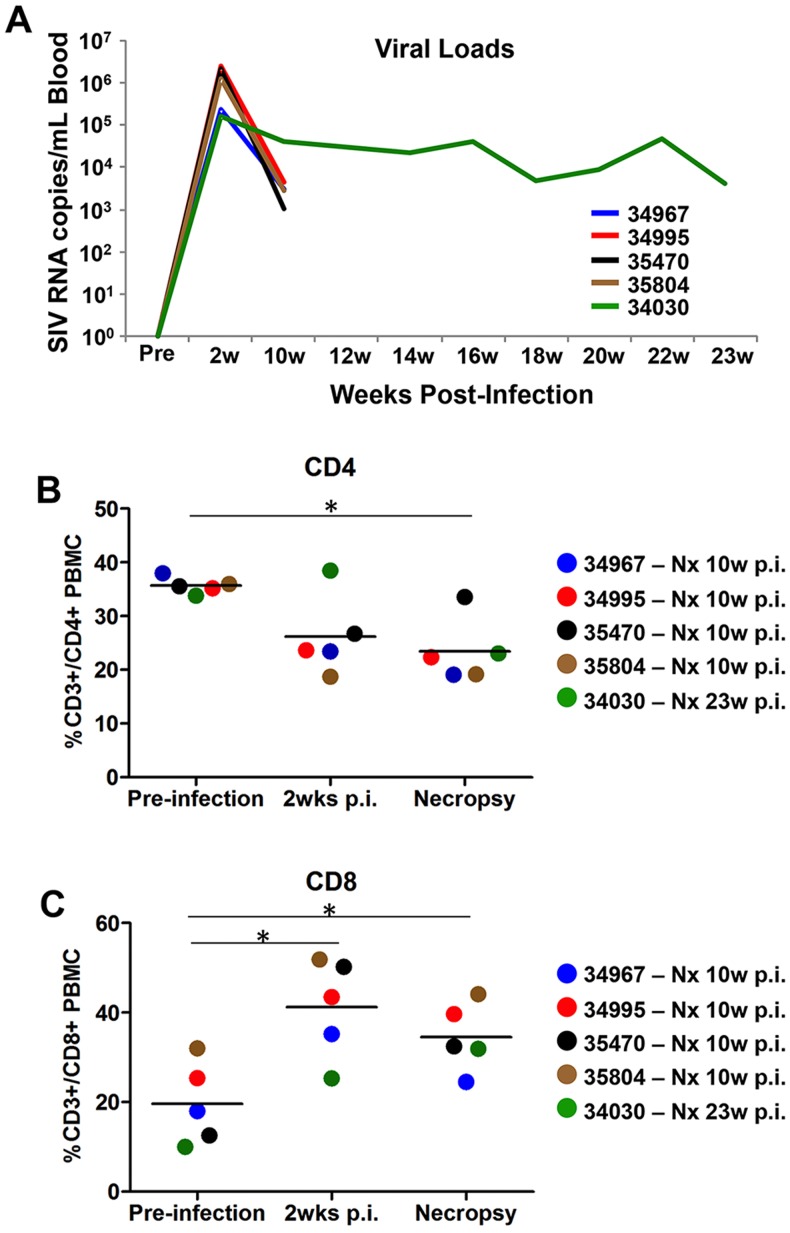
Clinical characteristics of chronically SIV infected rhesus macaques. A) Longitudinal analysis of SIV burden in the peripheral blood compartment of chronically infected macaques utilized in the study. B) Percentage of CD3+/CD4+ PBMC in macaques prior to SIV infection, and during acute (2 wks. p.i.) and chronic (10 wks. p.i., or 28 wks. p.i.) stage infection. C) Percentage of CD8+/CD4+ PBMC at the same time points. Note: * denotes *p*<0.05.

In light of the prevalence of oral manifestations in HIV infected patients and recent findings of dysbiosis in the oral microbiome [18], we wanted to determine if untreated chronic SIV infection was associated with changes in the resident bacteria on the tongue of rhesus macaques. All of the SIV infected macaques chosen for this retrospective study were inoculated intravenously with SIVmac251, received no antiretroviral therapy, and displayed normal levels of viremia during primary and chronic stage infection (Figure 3A). Four of the animals were necropsied after 10 weeks of SIV infection and one after 23 weeks. In comparison to healthy controls, CD4+ T cell percentages were substantially reduced in the blood of all but one of the SIV infected macaques by 2 weeks post-infection (p.i.) and were depleted with statistical significance for the entire group (*p*<0.05) at necropsy (Figure 3B). As expected, CD4+ T cell depletion from the blood was counterbalanced by a significant increase in the percentage of circulating CD8+ T cells (Figure 3C).

**Figure 4:**
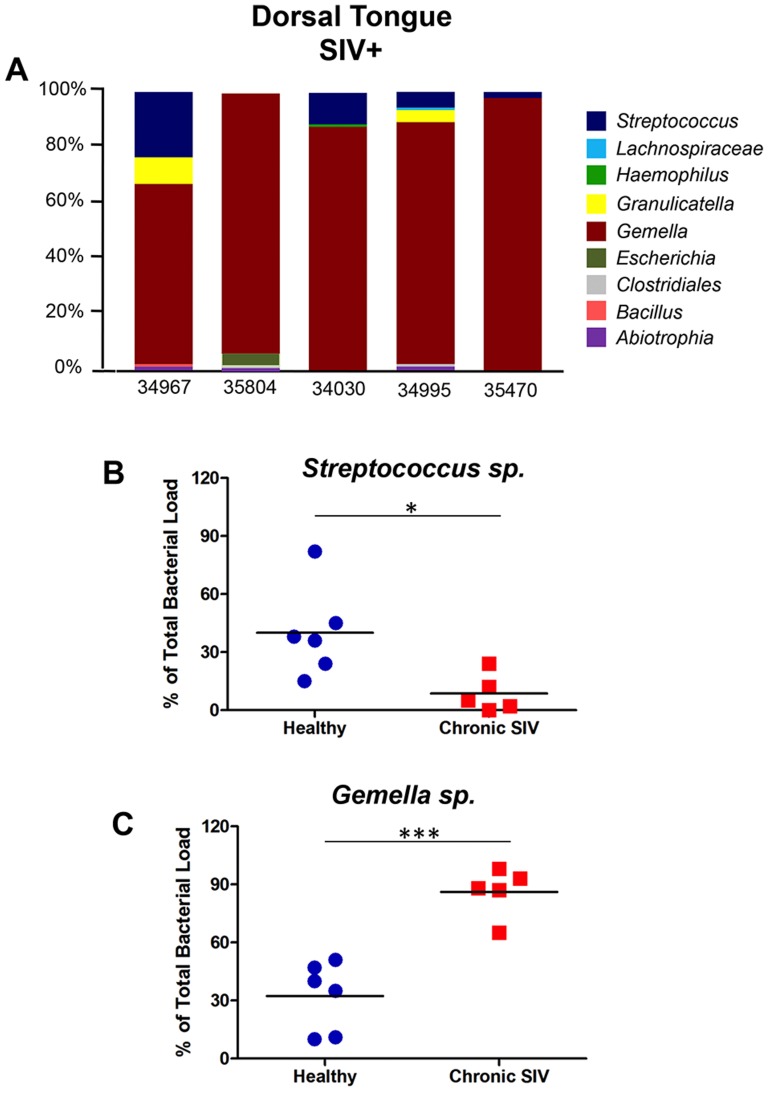
Dysbiosis of the lingual microbiome in chronic SIV infection. A) Abundance distribution of the bacterial genera colonizing the tongue dorsum of macaques with chronic untreated SIV infection. B) Comparison of the proportion of *Streptococcus spp*. in the lingual microbiome of healthy and SIV infected animals. C) Comparison of the proportion of *Gemella spp*. in the lingual microbiome of healthy and chronic SIV animals. Note: * and *** denote *p*<0.05 and *p*<0.001, respectively. *P*-values generated from one-sided Student *t*-test.

DNA was isolated from dorsal tongue tissues of SIV infected animals, and clonal analysis of the16S rRNA genes was utilized to compare bacterial communities to the communities observed in healthy uninfected animals. The lingual microbiome of SIV infected animals (Figure 4A) displayed a markedly different species profile than observed in healthy macaques (Fig. 2). Community diversity was reduced from a mean of 12 species in healthy animals to only 5 in the SIV infected group (*p*<0.01; data not shown). In addition, *Gemella spp.*, consisting primarily of *G. morbillorum* (human-colonizing), appeared to obtain a significant growth advantage over other all other commensal species (Figure 4B), comprising at least 79% of the total bacterial population in 4 of the 5 infected animals. The outgrowth of *Gemella* was contrasted by a statistically significant reduction in *Streptococcus spp.* growth (Figure 4C) that was consistent (albeit more dramatic) with our recent findings in HIV infected patients [18]. Interestingly, the outgrowth of *G. morbillorum* mirrored findings from a previous periodontal study of HIV infected subjects in which this species comprised an estimated 84% of the subgingival bacteria in one patient [19].

In addition to modulations in the proportion of core commensal bacteria, several potentially pathogenic species were detected on the tongue of SIV infected animals that were not found in any controls, including *Escherichia coli* (4% of total; macaque 35804) *Bacillus cohnii* (1% of total; macaque 34967), and a *Haemophilus aegyptius*-like bacterium (1% of total; macaque 34030). These data indicate that, similar to HIV infected humans [18], a potentially pathogenic footprint was established in the lingual microbiome during untreated chronic SIV infection, even in the absence of obvious opportunistic oral infections.

### Potential impact of host immune status

**Figure 5:**
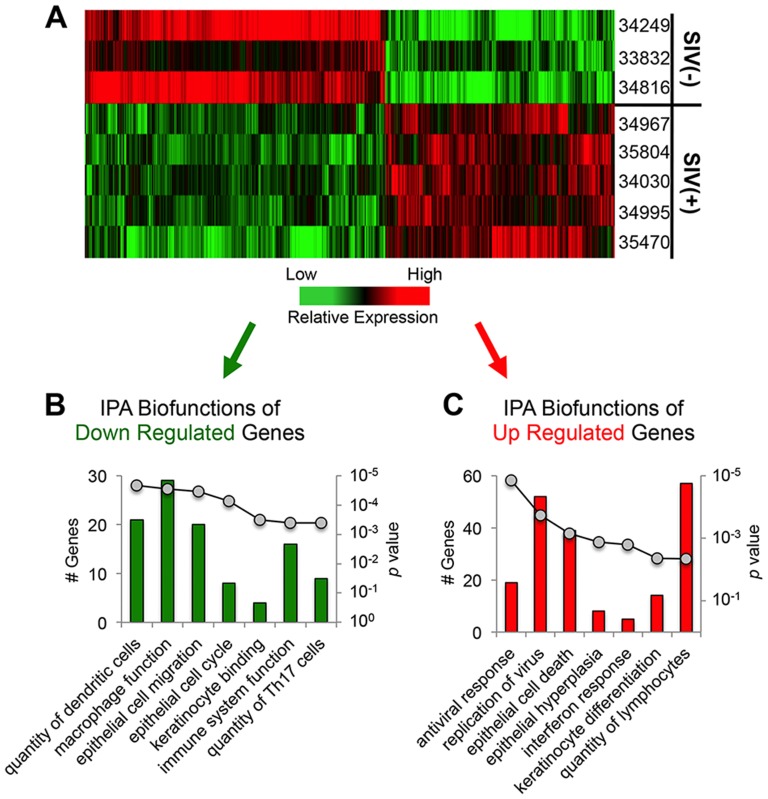
Gene expression profiling of the lingual mucosa in SIV infected macaques. A) Hierarchical clustering of genes differentially expressed (+/− 1.5 fold-difference and *p*<0.05) in the lingual mucosa of SIV infected animals and healthy controls as determined by microarray analysis. B) Pathway analysis of genes down-regulated in the lingual mucosa of SIV infected macaques. C) Pathway analysis of genes up-regulated in the lingual mucosa of SIV infected animals. Note: The number of genes in each functional category is shown in bar graphs on the primary y-axis and corresponding *p* values as a line graph on the secondary y-axis. Pathway analysis was performed with Ingenuity Pathway Analysis (IPA^©^) software.

We next analyzed host gene expression in tongue mucosa to evaluate potential links between alteration of the resident bacterial community and changes in immune function due to SIV infection. Transcriptional profiles of dorsal tongue tissues were compared between the 5 SIV infected animals and 3 healthy controls using Affymetrix rhesus macaque GeneChips©. Statistical analysis (fold-change +/− 1.5; p-value≤0.05) indicated a total of 905 genes were up-regulated in the SIV infected group and 1329 down-regulated (Figure 5A). Biofunctional analysis of the up-regulated transcripts using Ingenuity Pathway Analysis (IPA^©^) software revealed a statistically significant over-representation of transcripts associated with antiviral response, viral replication, epithelial cell death, epithelial hyperplasia, interferon response, keratinocyte differentiation, and lymphocyte quantity (Figure 5C).

**Figure 6:**
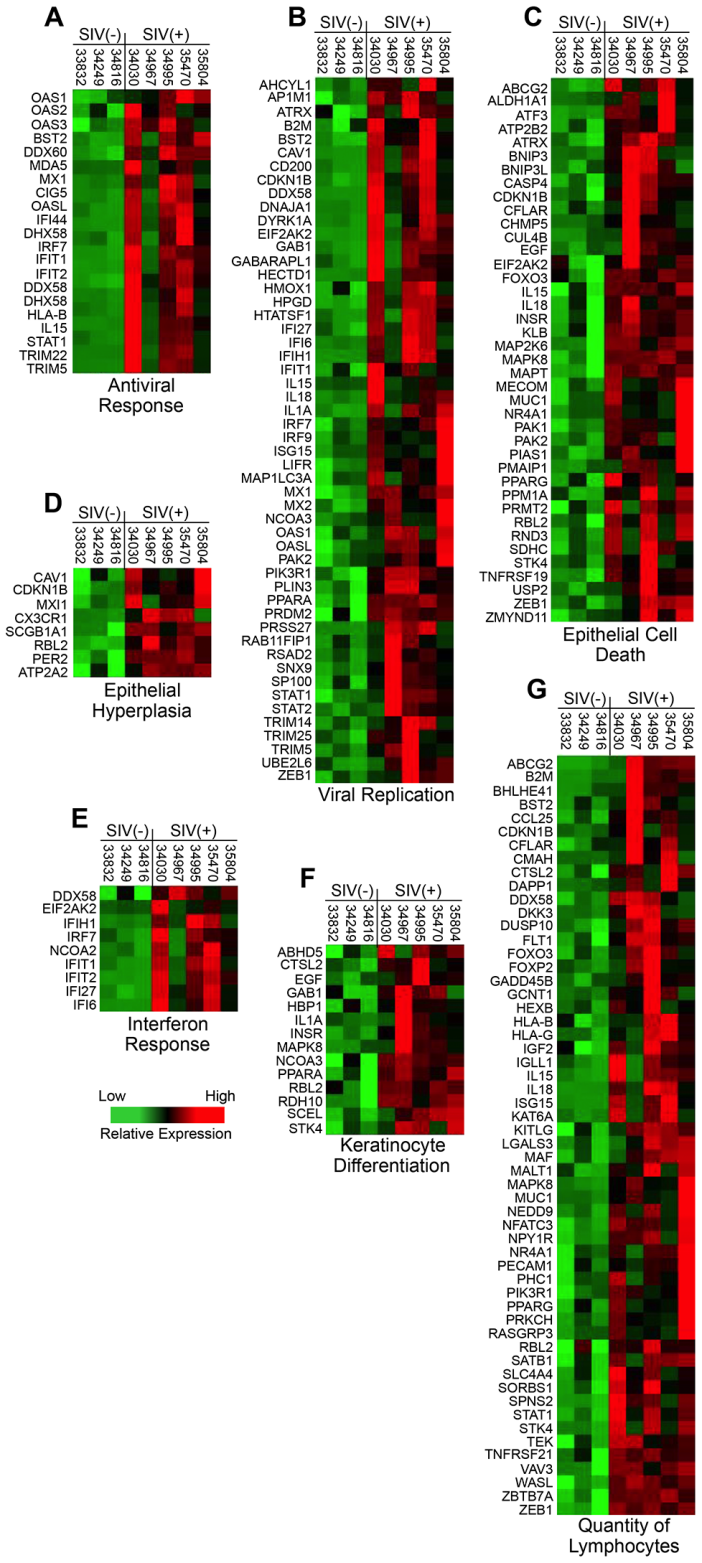
Transcriptional profile of genes in up-regulated functional categories. Heat maps are shown detailing the transcription levels of genes associated with A) antiviral response, B) viral replication, C) epithelial cell death, D) epithelial hyperplasia, E) interferon response, F) keratinocyte differentiation, and G) quantity of lymphocytes that were up-regulated in the tongue mucosa of SIV infected macaques.

Closer examination revealed that the antiviral responses (Fig. 6A) in the tongue encompassed a broad range of interferon (IFN) induced restriction factors that included tetherin (BST2), myxovirus protein-1, oligoadenylate protein-1, -2, and -3, and viperin (CIG5), as well as numerous molecules involved in viral replication (Fig. 6B) and IFN signaling pathways (Fig. 6E). Interestingly however, we were unable to detect significant quantities of SIV RNA in the tongue by RT-PCR or microarray assays. This apparent discordance may be indicative of response to ongoing localized SIV replication at undetectable levels, increased systemic antiviral signaling driven by uncontrolled SIV infection, and/or potential activation of endogenous viruses. Evidence for the later possibility is supplied by observations of increased transcription, in SIV infected animals, of genes from Cercopithecine and human herpesviruses for which probes are available on the rhesus macaque GeneChips (data not shown). Additional heat maps detailing the expression of genes up-regulated in each biofunctional category in the tongue mucosa of SIV infected animals are shown in Figure 6.

In light of the increased antiviral expression profile, it was also notable that the lists of up-regulated genes involved in epithelial cell death (Fig. 6C), keratinocyte differentiation (Fig. 6F), and lymphocyte quantity (Fig. 6G) included pro-inflammatory cytokines (IL-1A, IL-15, IL-18), chemokines (CCL25), and several other biomarkers of inflammation (MECOM, MUC1, PECAM1, PPARA, and PPARG). These findings are consistent with previous reports of the intestinal, lung, and vaginal compartments [34]–[36], and suggest that inflammatory responses were constitutively activated in the tongue mucosa during untreated chronic SIV infection. Presumably, unresolved inflammation and increased epithelial cell death on the tongue of SIV infected animals would have the potential to fundamentally alter the landscape available for host-microbe interaction. Supporting evidence for this possibility is provided by recent reports that chronic perturbations of IL-18 and CCL25 may be linked to alterations of the commensal microbiota [37], [38].

**Figure 7:**
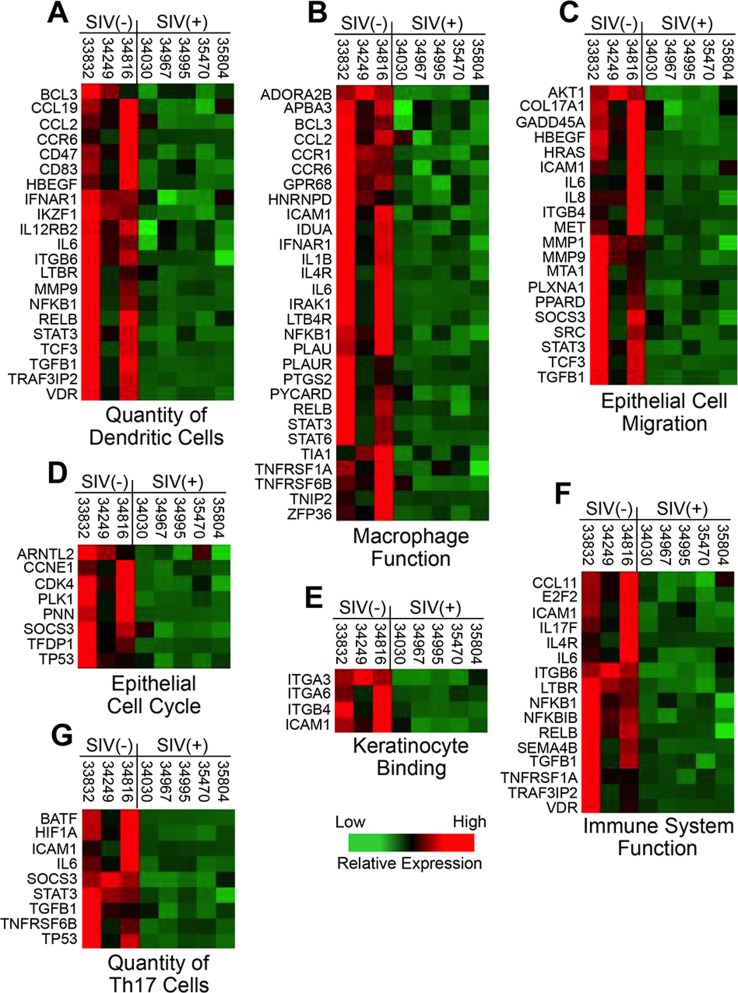
Transcriptional profile of genes in down-regulated functional categories. Heat maps are shown detailing the transcription levels of genes associated with A) quantity of dendritic cells, B) macrophage function, C) epithelial cell migration, D) epithelial cell cycle, E) keratinocyte binding, F) immune system function, and G) quantity of Th17 cells that were down-regulated in the tongue mucosa of SIV infected macaques.

The pathogenic implications of the up-regulated transcriptional profile in the lingual mucosa of SIV infected animals were also supported by functional analysis of genes that were down-regulated. Genes associated with dendritic cell quantity, macrophage function, overall immune system function, and several aspects of epithelial maintenance and regeneration (epithelial cell cycle, epithelial migration, keratinocyte binding, quantity of Th17 cells) were all transcriptionally repressed in the tongue (Figure 5B). We also noted that, in addition to the biofunctions identified by IPA analysis, several biomarkers of epithelial (keratinocyte) maturation, including stratifin, POU class 3 homeobox 1, involucrin, and basonuclin 1 were significantly suppressed below healthy baseline levels (data not shown). Thus, coupled with transcriptional evidence of increased inflammation and epithelial cell death (Fig. 6), the down-regulation of numerous genes associated with epithelial growth and maintenance provides additional evidence that mucosal barrier functions may have been compromised in SIV infected animals. Heat maps depicting the expression of genes that were down-regulated in each biofunctional category in the tongue mucosa are shown in Figure 7.

**Figure 8:**
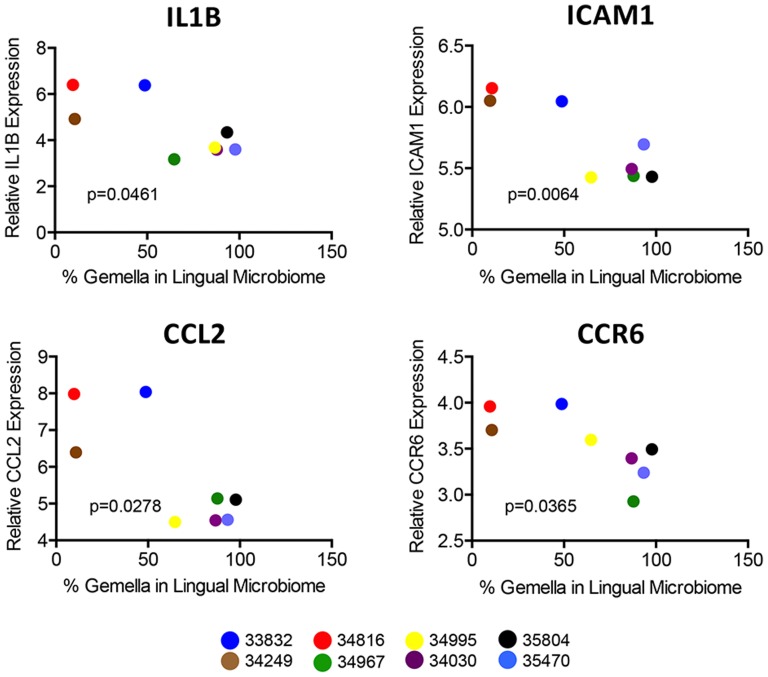
Correlation between expression of macrophage biomarkers and colonization of *Gemella morbillorum*. The relationship between the proportions of G. morbillorum in the lingual microbiome and the expression level of macrophage biomarkers IL1B, ICAM1, CCL2, and CCL6 in the lingual mucosa was evaluated in 9 macaques (3 uninfected and 6 SIV infected) by correlative analysis using Graph Pad Prism, version 6.0b. *P*-values are also shown for each correlation.

Given the potential impact of the loss of innate immune functions on host-microbe interactions, we were also intrigued by the down-regulation of biomarkers of macrophage function (Fig. 7A) and dendritic cell (DC) quantity (Fig 7B) in the tongue. The transcriptional profiles were supported by histopathologic assessments of lingual mucosal tissues that showed no discernable infiltration of DCs or monocytes/macrophages in infected animals compared to uninfected controls (data not shown). While any impairment in DCs and/or macrophage efficacy could have profound effects host-microbe homeostasis, the potential loss of macrophage function may have been particularly relevant to the outgrowth of *G. morbillorum* on the tongue. Previous reports from bone marrow transplantation studies in mice indicate that macrophages may play a primary role in controlling *G. morbillorum* colonization [39]. Interestingly, further evaluation showed that expression levels of 43% (13 of 30) of the down-regulated biomarkers of macrophage function correlated with the level of *Gemella* colonization on the tongue of all SIV infected animals and uninfected controls, including, IL-1B ICAM1, CCL2, and CCR6 (Figure 8).

**Figure 9:**
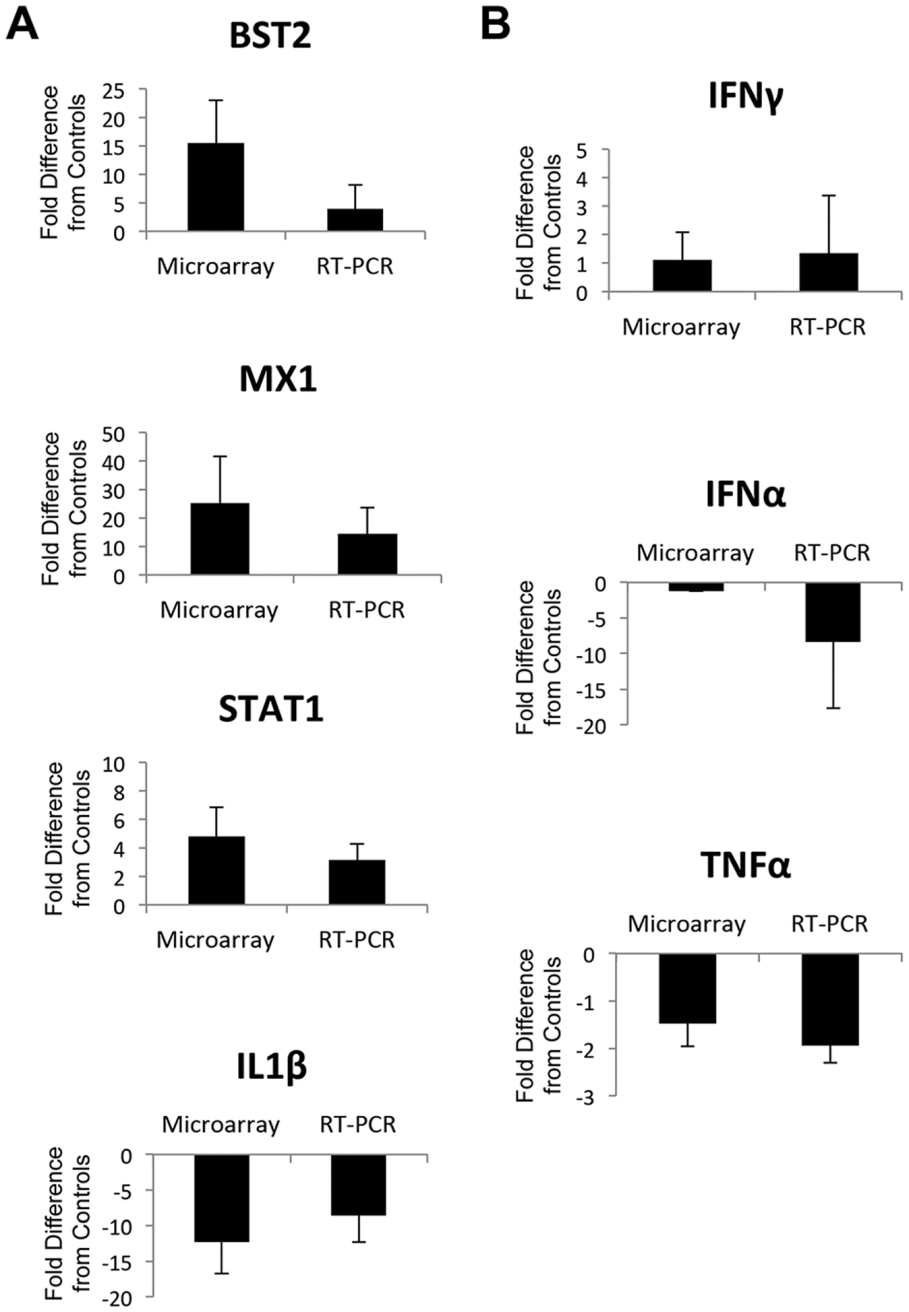
Validation of microarray data by quantitative real-time PCR. A) Comparison of fold changes in expression of BST2, MX1, STAT1, and IL1β in the tongue of SIV infected animals as determined by quantitative real-time PCR (RT-PCR) and microarray-based assays. B) Comparison of microarray and RT-PCR-based changes in expression of pro-inflammatory cytokines IFNγ, IFNα, and TNFα in the tongue of SIV infected macaques. Notes: error bars denote standard deviation of fold change in each system. RT-PCR data were generated from comparison of 3 SIV infected animals to 3 uninfected controls, and microarray data from comparison of 5 SIV infected animals to 3 uninfected controls.

To validate the microarray findings and provide additional supportive data, we next compared changes in the transcription of BST2, MX1, STAT1, and IL-1B that were detected by microarray to measurements obtained through quantitative real-time PCR (RT-PCR) assay (Figure 9A). The direction and level of fold-change detected for each of these genes were similar to the microarray findings. In addition, we utilized RT-PCR to evaluate transcription of 3 other key pro-inflammatory cytokines that showed intriguing differences by microarray analysis between infected and uninfected animals but did not reach the cutoff for statistical significance; IFN gamma (IFNγ), IFN alpha (IFNα), and tumor necrosis factor alpha (TNFα) (Figure 9B). Again, the trends observed in the microarray data were corroborated by RT-PCR analysis. IFNγ expression appeared to be constitutively induced in the tongue mucosa of SIV infected animals while IFNα and TNFα expression was repressed compared to baseline levels in healthy controls. These data suggest that the pathologic mechanisms established in the lingual mucosa during untreated chronic SIV infection may have been more influenced by activation of IFNγ signaling than signaling through IFNα or TNFα associated pathways.

### Potential role of epithelial pathogenesis in host-microbe dysbiosis

**Figure 10:**
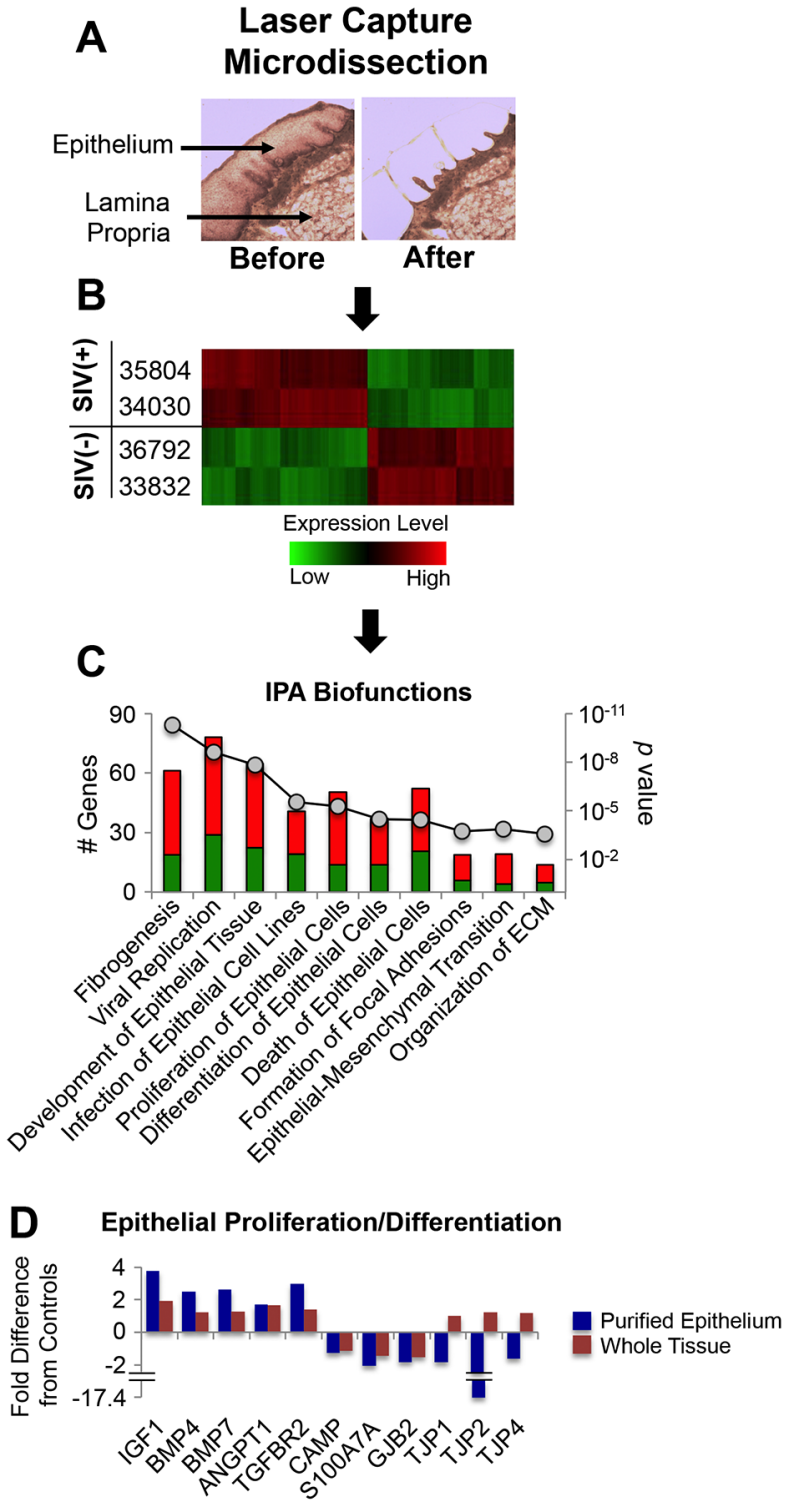
Transcriptional profiling of the macaque tongue epithelium. A) Representative images of rhesus macaque tongue tissue sections “before” and “after” laser capture microdissection of the epithelial layer. B) Hierarchical clustering of genes up- and down-regulated specifically in the tongue epithelium of SIV infected macaques (35804, 34030) in comparison to uninfected controls (36792, 33832). C) IPA^©^-based pathway analysis of genes up- and down-modulated in the tongue epithelium of SIV infected macaques. Statistically over-represented biological functions were identified in the list of differentially expressed genes in the tongue epithelium and are shown as stacked bar graphs with the contribution (# genes) from up-regulated transcripts in red and down-regulated transcripts in green. D) Comparison of fold changes in genes associated with epithelial proliferation and differentiation identified through analysis of epithelium purified by laser capture microdissection versus analysis of whole lingual tissues.

Evidence of an association between dysbiosis of the lingual microbiome and alterations in transcriptional programming of epithelial cells in SIV infected animals prompted us to evaluate the role of epithelial deterioration in greater detail. Utilizing laser capture microdissection (LCM), epithelial cells were isolated from lingual tissue sections of a subset of SIV infected animals and healthy controls (Figure 10A) and comparative analysis of transcriptomes was performed by microarray (Figure 10B). IPA-based biofunctional assessment of genes modulated in the epithelium of SIV infected macaques revealed a significant over-representation of factors mediating fibrogenesis, viral replication, development of epithelial tissue, infection of epithelial cell lines, proliferation, differentiation, and death of epithelial cells, focal adhesion formation, epithelial-mesenchymal transition, and organization of extracellular matrix (Figure 10C). A complete listing of the fold-changes and associated *p*-values for the genes assigned to each functional category is presented in Table S1. We found that many of the changes in transcription of epithelial biomarkers observed in analysis of whole lingual tissues were corroborated by assessment of purified epithelial cells. As shown in Figure 10D, this included an intriguing contrast between the expression of genes mediating growth factor signaling pathways and biomarkers of epithelial barrier maturation such as antimicrobial peptides (CAMP, S100A7) and tight junction proteins (GJB2, TJP-1, -2, -4), These data again underscore the likelihood that dysbiosis in the oral microbiome resulted, in part, from deterioration of the epithelial landscape.

**Figure 11:**
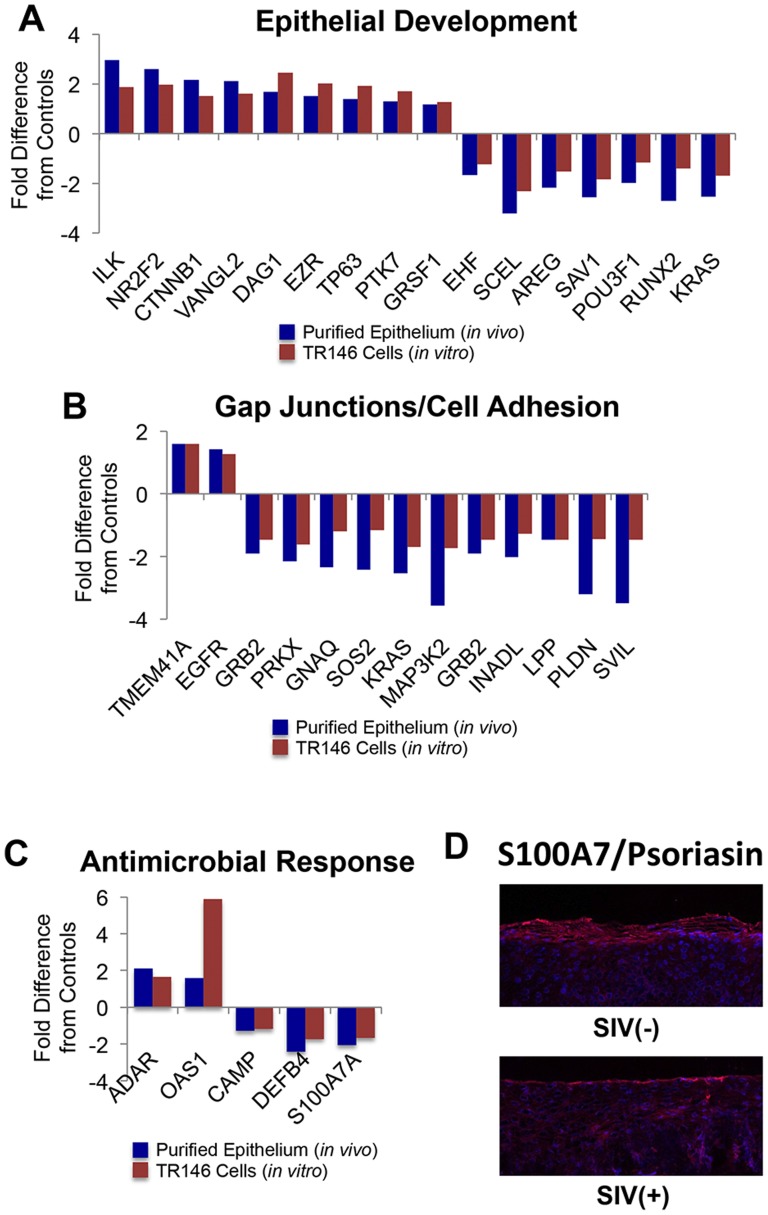
Impact of chronic IFNγ signaling on oral epithelial barrier functions. Comparison of *in vivo* and *in vitro* fold-changes in genes associated with A) epithelial development, B) gap junctions/cell adhesion, and C) antimicrobial response that were commonly regulated in the tongue epithelium of SIV infected macaques and in IFNγ treated TR146 oral epithelial cells. D) Immunohistochemical staining of psoriasin (red) protein in the tongue epithelium of a representative SIV infected macaque and healthy uninfected control. Cellular nuclei are counterstained with DAPI (blue).

Given evidence that increased IFN signaling in the tongue of SIV infected macaques (Figs. 6A, 6E) may have been associated with IFNγ stimulation (Fig. 9), we next explored the molecular impact of constitutive IFNγ signaling on epithelial functions under more controlled conditions utilizing the TR146 oral epithelial cell line. TR146 cells were grown to 90% confluence in tissue culture dishes before treating with either 20 mM IFNγ (experiment; n = 6), or a mock treatment (controls; n = 6) for 8 hours, and the resulting changes in gene transcription were assessed by microarray. The *in vitro* transcriptional changes in TR146 cells were then directly compared to the transcriptional profiles of the LCM isolated tongue epithelium to identify *in vivo* changes in SIV infected macaques that were likely to be driven by chronic induction of IFNγ signaling. This cross comparison revealed that several important factors involved oral epithelial development were similarly repressed in both *in vitro* and *in vivo* analyses including ets homologous factor, sciellin, amphiregulin, salvador homolog 1, POU class 3 homeobox 1, runt-related transcription factor 2, and v-Ki-ras2 Kirsten rat sarcoma viral oncogene homolog (Figure 11A). Moreover, multiple genes encoding gap junction proteins and other cell adhesion molecules were also down-regulated in both datasets (Figure 11B). The similarity between *in vivo* and *in vitro* expression profiles provide multiple lines of evidence that suggest chronic IFNγ signaling could have contributed to the impairment of epithelial development in the lingual mucosa of SIV infected animals.

Interestingly, while IFNγ treatment of TR146 cells resulted in the up-regulation of multiple interferon induced molecules and antiviral factors (e.g. OAS-1 and RNA-specific adenosine deaminase), genes encoding antimicrobial peptides (AMPs: CAMP, DEFB4, S100A7) were, in contrast, down-regulated (Figure 11C). Furthermore, this discordant pattern of expression in antiviral factors and AMPs was remarkably similar to the *in vivo* transcriptional profiles identified in LCM isolated tongue epithelium. We then validated the loss of S100A7/psoriasin indicated by microarray through comparative immunohistochemistry assays of SIV infected animals and uninfected controls (Figure 11D). It is also notable that, similar to humans [40], psoriasin was by far the most highly expressed AMP on the macaque tongue. Presumably, the down-modulation of multiple AMPs in the lingual epithelium also impacted the community structure of the commensal tongue microbiome, and may have contributed to the increased capacity of *E. coli* (Fig. 4) and other pathogenic species to gain a foothold.

## Discussion

Although oral infections are considered clinical biomarkers of HIV disease progression in developing countries, it is noteworthy that they are also often utilized to diagnose new HIV infections, suggesting that the establishment of dysbiosis in the oral cavity can be an early pathologic event. Multiple studies indicate that commensal oral bacteria can also escape from their normal niches and cause infective endocarditis, bacteremia, and septicemia [41], [42], and may do so at a higher frequency in HIV infected patients compared to healthy subjects. With regard to the current study, the commensal bacterium *Gemella morbillorum* has been reported in HIV patients as a putative etiological agent for periodontal disease [19], [43], endocarditis [44], and even fatal septic shock [45]. Our data demonstrate that *G. morbillorum* can gain an overwhelming growth advantage in the lingual microbiome during untreated chronic SIV infection of rhesus macaques. Indeed, given its well-documented role as an opportunistic pathogen [46]–[49], the magnitude of *Gemella* detection on the tongue of SIV infected animals might be more accurately described as a secondary infection rather than overgrowth of a commensal bacterium.

Interestingly, the loss of IL-1B and other of biomarkers of monocyte/macrophage recruitment in the tongue mucosa of SIV infected animals reveal a potential mechanism that could be exploited by *Gemella* to obtain a dominant foothold. Studies in the gastrointestinal tract indicate that macrophages are a primary source of IL-1B, and that commensal bacteria stimulate production of this cytokine [50]. Investigations of macrophage metalloelastase function in knockout mice (MME^-/-^) provide support for this conclusion, and further suggest that macrophages may be uniquely responsible for regulating *G. morbillorum* colonization [39]. Although we did not include MME (MMP12) in our gene lists because the *p*-value did not reach our criteria for significance (*p* = 0.21), it is notable that this transcript was down-regulated approximately 3-fold on average in the tongue mucosa of SIV infected animals, consistent with the down-regulation of other monocyte/macrophage lineage biomarkers (Fig. 5). Thus, our findings highlight the distinct possibility that compromised monocyte trafficking and/or macrophage function could be linked to the manifestation of *G. morbillorum* infections in HIV infected subjects.

In addition to the role of macrophages in regulating *Gemella*, epithelial pathologies, potentially driven by chronic IFNγ signaling also appear likely to negatively impact host interaction with the microbiome during chronic SIV infection. Previous studies have shown that IFNγ can play a large role in regulating the balance/rate of epithelial development [51]–[53], and suggest that constitutive induction may induce barrier deterioration [54]. In addition, studies of lingual inflammation in rodents show increased apoptosis in taste bud epithelia treated with IFNγ [55]. Our findings are in agreement with these reports, and further suggest that the impediment in epithelial defenses in SIV infection also manifests in reduced production of psoriasin and other AMPs that presumably regulate the microbiota. It is also worth noting that the bactericidal activity of psoriasin is highly specific to *E. coli* [56], raising the possibility that the loss of psoriasin may have contributed to the colonization of *E. coli* on the tongue dorsum of one of the SIV infected macaques (Fig. 4).

Laser capture microdissection-based analysis of tongue epithelial gene expression also revealed putative mechanisms of dysbiosis that may not be linked to IFNγ production (i.e. no concordance with IFNγ treated TR146 cells). These include down-regulation of genes associated with tight junction integrity (zona occludens-1 -2, -3, striatin; *p* value<0.05; Fig. 10D, Table S1, data not shown) and up-regulation of numerous collagen-encoding genes (COL1A1, 1A2, 3A1, 5A1, 5A2, 6A2, 7A1, and 8A2; *p* value<0.05; Table S1). The loss of tight junction expression provides further evidence that compromised barrier functions could allow translocation of oral bacteria during SIV (and HIV) infection. Increased collagen production, on the other hand, suggests that chronic fibrosis might act to physically alter the epithelial landscape and thereby affect interaction with certain commensal bacteria. Clearly, additional *in vivo* and *in vitro* studies will be necessary to fully define and understand the complex impact of HIV/SIV induced oral epithelial pathologies on host interaction with commensal bacteria, both individually and collectively within a biofilm.

Importantly, our findings support the continued development and use of rhesus macaques as a non-human primate model for investigations of oral manifestations of HIV disease. In sampling only 2 environmental niches of the macaque oral cavity we have identified 4 of the 5 phyla reported to comprise the “core oral microbiome” of healthy humans as determined through sampling of tongue, plaque, palate, cheek, and saliva [57]. Taken together, the data from our study shed new light on the oral biology of non-human primates and provide a framework for future SIV and HIV investigations to build upon. The SIV macaque model should prove to be an invaluable experimental tool for unraveling further details of the mechanisms that lead to dysbiosis and developing appropriate therapeutic strategies. In larger cohorts of HIV infected subjects, longitudinal studies based on non-invasive oral swab sampling might be very useful for determining the frequency and character of episodic dysbiosis and evaluating their connection to the establishment of secondary infections. In addition, preliminary studies to determine if opportunistic infections by *Gemella* or other oral bacterial species can be linked to impaired monocyte trafficking and/or macrophage function may be warranted.

## Supporting Information

Table S1 Fold-changes and associated *p*-values for genes up and down modulated in the epithelium of untreated chronically SIV infected macaques and biofunctionally assigned in Figure 10C by IPA^©^.
(XLSX)
